# Diffraction-free light droplets for axially-resolved volume imaging

**DOI:** 10.1038/s41598-017-00042-w

**Published:** 2017-02-07

**Authors:** G. Antonacci, G. Di Domenico, S. Silvestri, E. DelRe, G. Ruocco

**Affiliations:** 1 Center for Life Nano Science@Sapienza, Istituto Italiano di Tecnologia, Rome, Italy; 2 Department of Physics, University of Rome ”Sapienza”, Rome, Italy

## Abstract

An ideal direct imaging system entails a method to illuminate on command a single diffraction-limited region in a generally thick and turbid volume. The best approximation to this is the use of large-aperture lenses that focus light into a spot. This strategy fails for regions that are embedded deep into the sample, where diffraction and scattering prevail. Airy beams and Bessel beams are solutions of the Helmholtz Equation that are both non-diffracting and self-healing, features that make them naturally able to outdo the effects of distance into the volume but intrinsically do not allow resolution along the propagation axis. Here, we demonstrate diffraction-free self-healing three-dimensional monochromatic light spots able to penetrate deep into the volume of a sample, resist against deflection in turbid environments, and offer axial resolution comparable to that of Gaussian beams. The fields, formed from coherent mixtures of Bessel beams, manifest a more than ten-fold increase in their undistorted penetration, even in turbid milk solutions, compared to diffraction-limited beams. In a fluorescence imaging scheme, we find a ten-fold increase in image contrast compared to diffraction-limited illuminations, and a constant axial resolution even after four Rayleigh lengths. Results pave the way to new opportunities in three-dimensional microscopy.

## Introduction

The ability of an optical system to image objects inside a full three-dimensional environment is ultimately dictated by diffraction, absorption and random scattering, all being especially important in biological systems. Diffraction forces high-resolution imaging to involve light fields with large apertures, so that working in the volume forcibly involves impractically large launch and detection optics. Absorption and scattering, in turn, limits light penetration. At present, standard imaging methods in the visible can only penetrate a few hundreds of micrometers inside a biological tissue^1^. For example, diffusive scattering inhibits the use of confocal microscopy, with its characteristic high axial resolution and optical sectioning, to analyze deep regions of a turbid sample^2^. Over the last few years, the demand for innovative imaging techniques that provide quantitative information in deeper regions of biological tissues has strongly increased. Besides infrared^3^ and ultrasound methods^4^, which are limited by a low spatial resolution, several techniques have been developed to obtain fast volumetric imaging using visible light. For example, optical coherence tomography (OCT) uses low coherence interferometry to perform tomographic reconstruction of internal tissue structures^5^ and has been recently shown to enable a sub-micron resolution^6^. Nevertheless, its ability to penetrate deep into a sample still depends on the wavelength of the laser source.

Nonlinearity can expand arbitrarily the depth of focus of an imaging system but requires the use of specific hosting materials^7, 8^. Bessel beams, in turn, provide a significant increase in the penetration depth without nonlinearity. These are non-diffracting beams of light resulting from the interference of a set of plane waves whose wave vectors form a cone^9^. Although the existence of pure Bessel beams requires an infinite amount of power, a similar approximate behaviour is achieved using finite annular pupils illuminated by Gaussian beams, the so-called Bessel-Gauss beams^10^. These have been used in imaging, such as depth imaging fluorescence microscopy^11^, light-sheet^12^ and light-field microscopy^13^, to enable imaging of live-cell dynamics^14^, zebrafish embryos^15^, and neuronal activity^16^. Bessel beams have also been studied for material processing^17^ and optical tweezing and trapping^18–20^. Ironically, it is this very non-diffracting nature with its enhanced penetration that makes them unsuitable for imaging in basic schemes, where an extended depth of focus naturally washes out axial resolution.

In our experiments, we achieve non-diffracting light fields with an effective finite depth of focus, comparable to that of a Gaussian beam. The apparently counterintuitive property emerges in the form of a non-diffracting spatial sequence of three-dimensional spots of light, that we term light droplets. To describe the basic idea behind the droplets we recall that a zeroth-order Bessel beam is formed by the monochromatic superposition of plane waves that propagate at a fixed angle *θ* with respect to a given axis $\hat{z}$. Since the waves share the same wavevector component ${k}_{\parallel }=k\cdot \,\cos \,\theta$, where *k* = 2*π*/*λ* and *λ* is the wavelength, there are no phase shifts along the axis between the interfering plane waves. This gives the beams a *z*-invariant filed distribution

$$E(\rho ,z)={J}_{0}({k}_{\perp }\rho )\exp (j{k}_{\parallel }z),$$

where *J*_0_ is the zeroth-order Bessel function and $\rho =\sqrt{{x}^{2}+{y}^{2}}$ is the distance from the beam axis in the transverse *x*,*y* plane. To introduce *z*-dependence but preserve the diffraction-free and self-healing properties, we superimpose *n* coaxial Bessel beams, each with a distinct value of *θ*_*i*_ (*i *= 1, …, *n*). The result is the interference light-droplet structure $I{(\rho ,z)}_{n}={J}_{0}{({k}_{\perp }\rho )}^{2}{|{\sum }_{i\mathrm{=1}}^{n}\exp (jk\cos {\theta }_{i}\cdot z)|}^{2}$.

**Figure 1:**
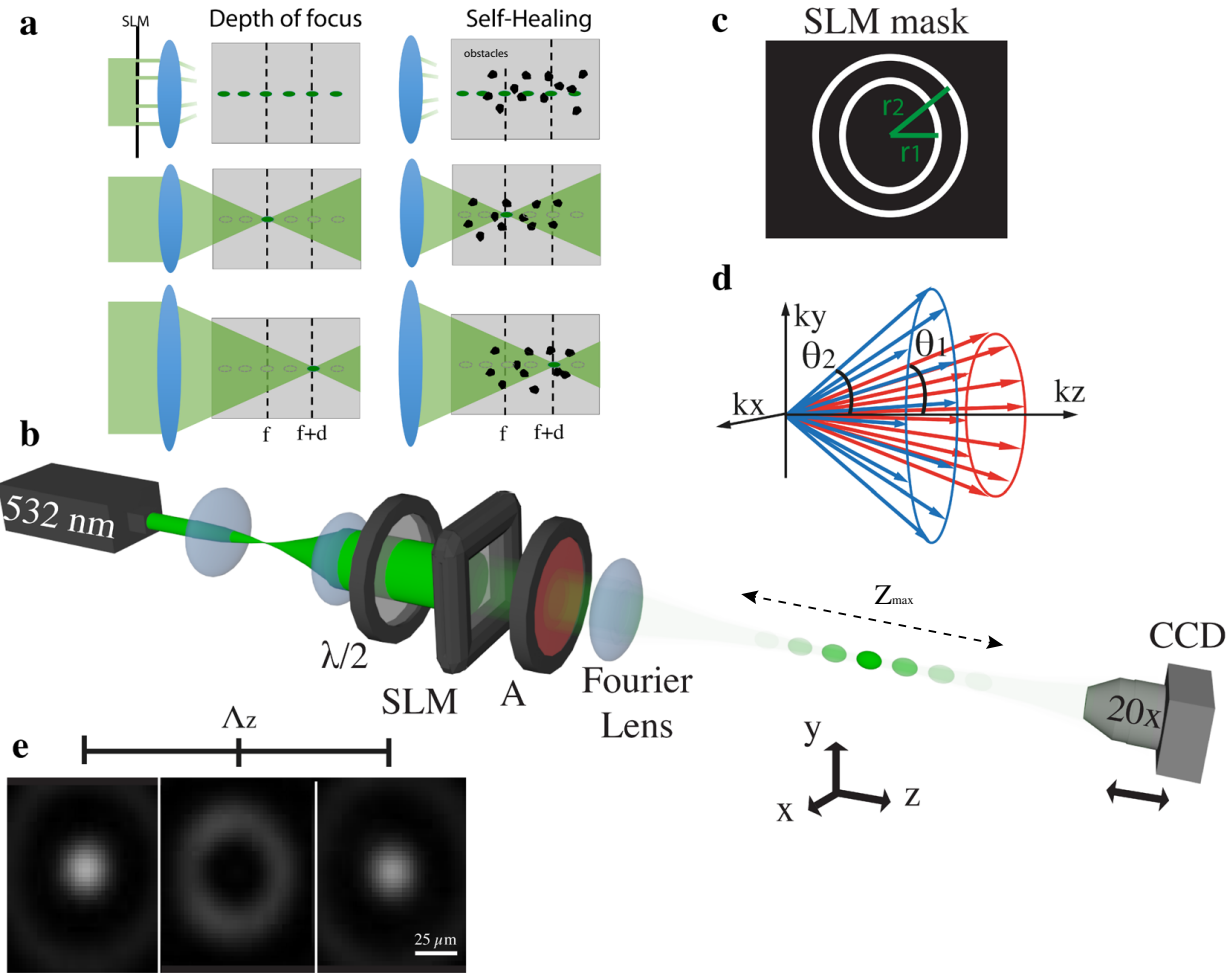
Schematic of a light droplet illumination in contrast with a standard Gaussian configuration (**a**). A Gaussian beam can illuminate a single voxel at different depths in a sample volume through lenses of different focal lengths. In turn, light droplets are periodic structures which enable a localised illumination along the whole optical axis. Optical setup diagram (**b**). A binary mask (**c**) displaying two concentric annuli of different diameters is generated on the SLM. This operates in amplitude mode through a *λ*/2 plate and a crossed analyser (A). The resulting field distribution gives rise to two co-propagating Bessel beams of different *k*-vectors (**d**). The beams are recorded through a 20x objective lens and a CCD camera. Transverse intensity distributions at three different positions along a period of Λ_*z*_ = 4 mm (**e**).

Figure 1 shows a schematic of the principle behind our light droplets (Fig. 1a) and a diagram of the optical setup for a droplet structure with *n* = 2 (Fig. 1b). A Spatial-Light-Modulator (SLM) is used to modulate the amplitude of an expanded laser beam (532 nm) using a binary mask with multiple concentric annuli of different radii *r*_1_ and *r*_2_ (Fig. 1c), these determining the corresponding *θ*_i_ and the associated *k*-vectors (Fig. 1d). The droplet field is now generated using a spherical lens that performs a Fourier transform of the field distribution at the pupil plane where two co-propagating Bessel beams with *θ*_1_ ≠ *θ*_2_ beat along the $\hat{z}$ axis.

The three-dimensional intensity distribution is investigated imaging the beam profile for each plane along $\hat{z}$ through a 20x objective lens onto a CCD camera. To maintain a constant magnification of the beam profile, measurements were performed by translating both the objective lens and the CCD camera along the $\hat{z}$ direction. Three planes are reported in Fig. 1e showing how the central light spot turns dark and bright along the $\hat{z}$ axis as a result of the destructive and constructive interference between the two beams (the sequence of droplets). Both the radius *r*_1_ and *r*_2_ of the annuli of the mask and the numerical aperture (NA) of the Fourier lens ultimately fix the propagation angles *θ*_1_ and *θ*_2_ of the interfering wavefronts, which in turn sets the period length of the light droplets in the $\hat{z}$ direction to ${\Lambda }_{z}=2\lambda /|\,\cos \,{\theta }_{1}-\,\cos \,{\theta }_{2}|$. Since the maximum propagation distance for Bessel beams is ${Z}_{{\rm{\max }}}=R/\sin (r/f)$, with *R* and *f* being the radius and the focal length of the Fourier lens^9^, the maximum number of droplets is given by $N={Z}_{{\rm{\max }}}/{\Lambda }_{z}\simeq R|\,\cos \,{\theta }_{1}-\,\cos \,{\theta }_{2}\mathrm{|/[2}\lambda \,\sin ({r}_{2}/f)]$.

**Figure 2:**
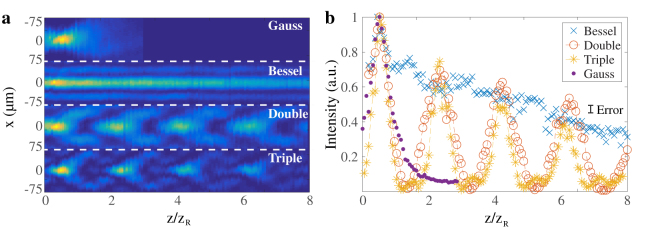
Top view (**a**) and axial intensity profile (**b**) of Gaussian, Bessel beams (*n* = 1) and light droplets obtained with a double (*n* = 2) and a triple annular (*n* = 3) mask respectively. Whereas the Gaussian beam diverges after the lens focus, both Bessel beam and light droplets are shown to be non-diffracting.

Figure 2 reports the beam intensity profiles along the $\hat{z}$ axis for a Gaussian beam and different light-droplet realizations. Distances are normalised to the Rayleigh length ${z}_{R}=\pi {\rm{n}}{w}_{0}^{2}/\lambda$, with *w*_0_ being the minimum beam waist of the Gaussian beam at the focal plane. In Fig. 1a the beams were obtained using a Gaussian mask, a single annulus (*n *= 1), two annuli (*n *= 2), and three concentric annuli (*n *= 3). Unlike the diffracting Gaussian beam, which diverges after the focal plane of the Fourier lens, the Bessel beam propagates along the optical axis with a constant width of the central peak. Diffraction-free light droplets arise from the interferometric beating of co-propagating Bessel beams for *n *> 1. Figure 2b reports the *x*-*y* integrated intensity distribution of the beams along the $\hat{z}$ axis. Light droplets in the *n *= 3 case show a more localized profile in $\hat{z}$ compared to the *n *= 2 case, as expected from the three wave interference. For a fixed sample thickness and experimental scenario, a sufficiently high value of *n* will, in principle, give rise to a single diffraction-free light droplet.

**Figure 3:**
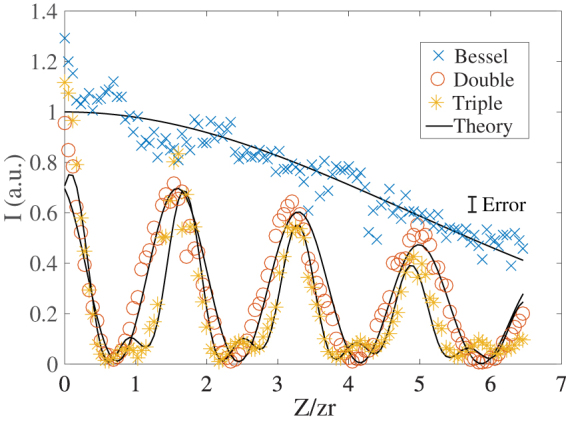
Experimental data of the normalised beam intensity and their theoretical prediction (full curves) in the case of a simple Bessel-Gauss beam (*n* = 1) (blue circles), two Bessel-Gauss beams (*n* = 2) (red circles) and three Bessel-Gauss beams (*n* = 3) (green circles).

Figure 3 illustrates the normalised peak intensity of a Gauss-Bessel beam and light droplets (*n *= 2, 3) measured along the $\hat{z}$ direction. The data shows good agreement with the theoretical predictions described in the Methods.

**Figure 4:**
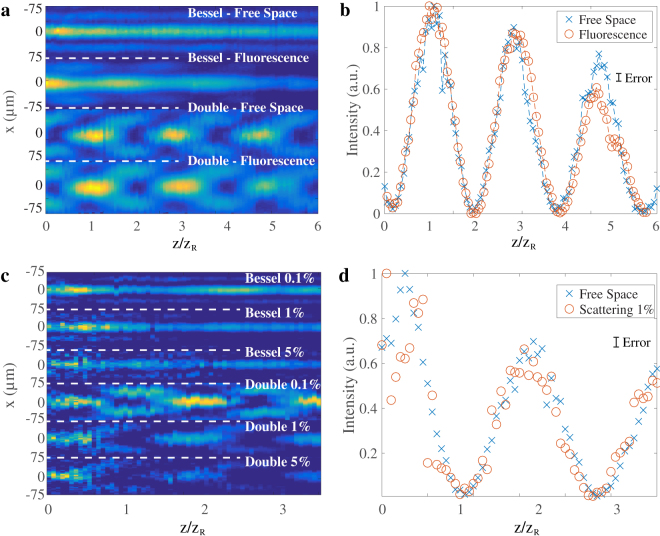
Top view (**a**) and axial intensity profile (**b**) of the fluorescent signal given by the light droplet illumination. A thin glass layer labeled with *Rhod-2* fluorescent molecules was used as a sample. To maintain a constant magnification, the glass layer was translated along the $\hat{z}$ direction together with the imaging system. As expected, the fluorescent signal reproduces the illumination profile. Top view (**c**) and axial intensity profile (**d**) of light droplets in water-milk solutions at different concentrations. The beams are shown to form droplets also in the presence of absorption and scattering.

In Fig. 4a,b we demonstrate the use of light droplets to excite and detect fluorescent molecules typical of most imaging schemes. We measured the fluorescent emission given by the *Rhod-2* fluorescent dye labelling a thin glass layer. In Fig. 4a we report the resulting fluorescent signal given by both Bessel beam and light droplet illuminations along the optical axis. The labeled glass layer was first imaged at the CCD plane and then translated along the $\hat{z}$ direction together with both objective lens and CCD camera to maintain a constant magnification during the scan. A long pass filter was placed at the detector plane to reject all the incident light transmitted by the glass and to detect only the fluorescent signal emitted, which had a central wavelength at approximately 581 nm.

In Fig. 4c,d we demonstrate light droplets in strongly scattering liquid volumes. We report the intensity distribution of light droplets compared to simple Bessel beams in water-milk solution at concentrations that simulate real biological tissues (water-milk scattering is similar to that of intralipid solutions in the range of 1% and 10%)^21^. All beam profiles were taken along the propagation in a glass cuvette of 10 mm length filled with the scattering liquid. Despite a decreasing signal strength and contrast, both Bessel beams and light droplets maintain equal profile and propagation path as the milk concentration increases.

**Figure 5:**
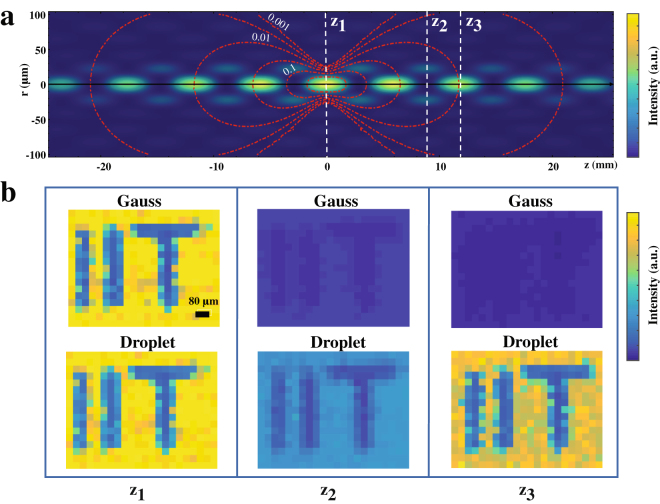
Top view of the droplet intensity distribution and contour (red dashed) lines of a focused Gaussian beam (**a**). A set of images were acquired at three different z-positions along the illumination axis (**b**). Images acquired at the focal plane (*z *= *z*_1_) with both Gauss and droplet illuminations exhibited equal contrast. However, as the sample was axially displaced from the focal plane, the contrast given by the Gaussian illumination rapidly dropped to zero, whereas the droplet illumination gave a decrease (*z *= *z*_2_) followed by a 10x-increase (*z *= *z*_3_) in the image contrast, the manifestation of the droplet axial-localization.

In Fig. 5 we use light droplets in a standard confocal imaging system. We report a set of images acquired in transmission mode with both Gaussian and droplet illuminations. In particular, a customized fluorescent sample was scanned in the $\hat{x}$ and $\hat{y}$ directions across the three different z-planes illustrated in Fig. 5a. As expected, both Gaussian and droplet images show equal contrast at the focal plane of the illumination lens (*z *= *z*_1_), as shown in Fig. 5b. Images obtained with the Gaussian beam illumination exhibit the natural sharp diffraction-induced decrease in the imaging contrast as the sample is axially displaced from the illumination focal plane. In turn, the images acquired with the droplet illumination exhibited a significantly reduced contrast near the local droplet minimum (*z* = *z*_2_) followed by a sharp increase at *z* = *z*_3_, where the signal-to-noise-ratio was measured to be more than 10-fold higher than that given by the Gaussian illumination. Unlike Bessel beams, the finite depth of field of the droplets provides a high z-localization in deep regions of a sample.

Our results demonstrate that light droplets allow selective multi-point 3D localized illumination inside volumes. Ideally, mixing more Bessel beams (*n* > 3) can lead to stronger axial localization, this at the expense of a more complex mask. However, as already evident in our *n* = 3 case, the interference pattern along the axis becomes more complicated, with secondary beating spots, and this ultimately leads to a decreased effective axial visibility and hence axial resolution. Axial localization is a key factor in 3D fluorescence microscopy, where it provides higher imaging contrast given by a more efficient photon balance along the optical axis and minimizes background noise outside the excitation region, which in turn limits the photobleaching of the fluorescent molecules at a minimal level. Furthermore, results demonstrate that the droplets maintain their spatial profile as well as their propagation path in turbid liquids, a feature that, as known to occur for conventional Bessel beams^22^, can partially overcome diffusive scattering typical of biological tissues, potentially allowing *in vivo* imaging of thicker, more densely populated fluorescent specimens in a backscattering configuration. Self-adaptive and phase-conjugation techniques can be applied to further overcome the effect of scattering on both signal strength and contrast^23^. A ten-fold increase in the imaging contrast and a higher axial localisation were demonstrated in a fluorescence imaging scheme. As such, our light droplet illumination may be applied to the other spectral-imaging techniques that rely on confocal schemes^24, 25^.

Since the droplets result from the interference of Bessel beams, introducing a relative phase modulation between the annuli will shift the pattern along its axis. Furthermore, the direction of the droplet illumination can be changed shifting the center of the annuli in the Fourier plane. As a result, motionless scanning data acquisition of three dimensional images could be achieved using a combination of amplitude and phase modulation in the SLM-Fourier plane. In turn, our droplet illumination could be used, for example, in optogenetics to study the activity of three-dimensional neuronal networks, where single neurones could be selectively activated without the need of mechanical movements. Furthermore, temporal switching between single light droplets could be obtained from the beating of multiple Bessel beams generated by light sources of slightly different wavelengths.

## Methods

### Experimental Setup

A CW laser working at 532 nm was used in all experiments. The beam was expanded at a magnification of 40x to illuminate a transmissive SLM (Holoeye, LC2012), which had a pixel pitch of 35 *μ*m and a fill factor of 56%. To set the SLM in amplitude mode, the polarization of the incident laser was set at 45° to the $\hat{y}$ axis by a *λ*/2 plate, and a crossed analyzer was placed after the SLM. A spherical lens of *f* = 200 mm focal length was placed next to the analyzer to perform a Fourier transform of the amplitude beam distribution. With a Gaussian beam illumination, this gave a minimum beam waist of *w*_0_ = 24.2 *μ*m and a Rayleigh length of *z*_*R*_ = 3.5 mm. To obtain the Bessel beam, a single annulus of *r*_1_ = 0.8 mm radius and 150 *μ*m width was displayed on the SLM, whereas for the droplets a second annulus of *r*_2_ = 2.8 mm radius was added to the mask, as shown in Fig. 1b. These two annulus radii set the droplet period Λ_*z*_ described above. More localized droplets were obtained by adding a third annulus of *r*_3_ = 4.1 mm radius, which was chosen so as to keep the same period given by the two-annuli case. All beams were imaged by a 20x objective lens (Olympus, UplanS) and a CCD camera (Thorlabs, DCC1545M) with an equal data acquisition time in each set of measurements. Three dimensional profile reconstruction was performed by translating the imaging system constituted of both objective lens and CCD camera by a mechanical stage with step size of 200 *μ*m.

### Data Processing

A two-dimensional Gaussian fit was performed on each frame to find the central peak location of the Bessel beams. We assumed this position to be equivalent to that of light droplets acquired next to the Bessel beams. All plots were normalised on the $\hat{z}$ axis to the Rayleigh length given by the Gaussian beam illumination. One-dimensional beam profiles along $\hat{z}$ were obtained by integrating values of 50 pixels associated to the intensity of the central peaks.

### Theoretical Analisys

According to the Gori-Guattari theory^10^, the field distribution of a Bessel-Gauss beam is given by

1 $$E(r,z)=A\frac{{w}_{0}}{w(z)}\,\exp \,[i((k-\frac{{\beta }^{2}}{2k})z-\Phi (z))]\\ \quad \quad \quad \quad \times {J}_{0}[\frac{r\,\beta }{\frac{i\,z}{{z}_{R}}+1}]\,\exp \,[(\frac{i\,k}{2\,R(z)}-\frac{1}{{w}^{2}(z)})({r}^{2}+\frac{{\beta }^{2}{z}^{2}}{{k}^{2}})],$$

where *A* is an amplitude factor, $w(z)={w}_{0}\sqrt{1+{(z/{z}_{R})}^{2}}$ is the beam waist along the propagation, *β* = *k* sin(*θ*) is the component of *k* orthogonal to the propagation direction at a given angular half-aperture *θ* of the cone, $R(z)=z\mathrm{/(1}+{z}_{R}^{2})$ is the radius of curvature of the beam wavefronts and $\Phi ={\tan }^{-1}(z/{z}_{R})$ is the Gouy phase shift. Equation 1 generalizes both Gaussian and Bessel field solutions, where *E*(*r*,*z*) reduces to a Gaussian field for *θ* = 0, and to a Bessel field for *w*_0_ = 0. Experimental data in Fig. 3 was fitted using the intensity distribution given by $I(r,z)=|{\sum }_{i\mathrm{=1}}^{n}{E}_{i}(r,z{)|}^{2}$, where *n* = 1 reduces to the standard case of a Bessel-Gauss beam, and *n* = 2, 3 gives solutions for the light droplets. As expected for Bessel-Gauss solutions, the peak intensity of single droplets decreases along $\hat{z}$.
